# Toxicology and Biocompatibility of Nanomaterials

**DOI:** 10.3390/nano11113110

**Published:** 2021-11-18

**Authors:** Carsten Weiss, Silvia Diabaté

**Affiliations:** Institute of Biological and Chemical Systems-Biological Information Processing, Karlsruhe Institute of Technology (KIT), Hermann-von-Helmholtz-Platz 1, 76344 Eggenstein-Leopoldshafen, Germany; silvia.diabate@kit.edu

It is our great pleasure to introduce this Special Issue entitled “Toxicology and Biocompatibility of Nanomaterials”. As the understanding of materials at the nanoscale and the ability to control their structure improves, a wide range of nanomaterials (NMs) with novel characteristics and applications are being fabricated for electronics, engineering, and, more recently, biomedical research applications. Although the technological and economic benefits of NMs are obvious, concern has also been raised that the very same properties, which enable a variety of novel applications, might have adverse effects if such a material is inhaled, ingested, applied to the skin or even released into the environment [1]. These concerns have led to an increasing discussion worldwide about possible regulatory policies for NMs. Therefore, there is a clear need to establish convincing scientific knowledge to assess the impact of NMs on human health and the ecosystem. These questions can only be tackled by collaborative research at the interface of engineering, physics, chemistry, toxicology and biology, as outlined in our first Special Issue on the topic one decade ago [2]. Meanwhile, the field of nanotoxicology has come of age, and is an established discipline in toxicology.

This Special Issue comprises six research articles and two timely reviews and covers research in the field of nanotoxicology, with a particular interest in molecular mechanism of action as well as the safe-by-design concept, i.e., the synthesis of biocompatible nanomaterials. Additionally, the impact of the biomolecular corona, which is the interaction of biomolecules with the NM surface, on toxicity and biocompatibility, is addressed. 

The first study addresses the effects of ZnO NMs on blood glucose levels in healthy and diabetic rats and discusses potential clinical applications [3]. The following articles are mainly in vitro studies with a focus on mechanisms of NM toxicity. Macrophages are important targets of NMs; therefore, the understanding of molecular initiating events is of high relevance. A series of TiO_2_ NMs with different characteristics were studied in rat alveolar macrophages and activation of the NLRP3 inflammasome is presented, highlighting the importance of case-to-case studies for proper hazard assessment [4]. In murine macrophages, the poorly understood mixture effects of co-pollutants and NMs has been investigated [5]. The authors demonstrate synergistic activities of silica NMs and genotoxic agents, thus reinforcing the notion that there is an urgent need to pay more attention to mixture effects in the future. Apart from the innate immune system, the gastrointestinal tract is an important target tissue. Therefore, the toxicity of silica NMs is explored in gastrointestinal cells [6], specifically in the presence or absence of serum, because the biomolecular corona has previously been shown to critically determine detrimental effects in other cell types [7]. Although in the presence of serum even pro-proliferative effects have been shown in gastric cells [8], in the absence of serum, silica NMs potently induce cell death in colon carcinoma cells; however, this is independent of the key regulators p53 and BAX, suggesting the potential for their further development as anticancer nanodrugs [6]. Although the field of nanotoxicology has matured and solved most of the initial technical problems and challenges, there are still issues which hamper proper hazard assessment. These include, but are not limited to, the reproducible synthesis of NMs with clearly characterized physico-chemical properties [9], as well as the establishment of more physiologically relevant test systems [10] and standard operating procedures for toxicity testing to provide comparable results across laboratories. Thus, fifteen European laboratories performed an inter-laboratory comparison to assess the toxicity of polystyrene NMs with the widely used MTS assay and provide guidance on how to improve reliable testing [11]. Finally, the interactions of CeO_2_ and TiO_2_ NMs and algae were addressed in an ecotoxicity study, and the importance of the adherence of NMs to the test organism was identified as an important parameter to predict toxicity [12].

Two reviews conclude this Special Issue. The first summarizes our knowledge of airborne NMs and their potential adverse effects on the nervous system with a specific focus on neurodegenerative diseases [13]. The final contribution provides a fresh outlook on positive aspects of NM actions in biological systems, i.e., their use in medicine as tools to diagnose and treat cancer [14].

In conclusion, we would like to thank all the authors for their interesting and excellent contributions, and the many constructive reviewers and the editorial team who helped to bring this Special Issue to fruition. We hope that a broad readership will enjoy this Special Issue.

## Data Availability

Not applicable.
